# Potential underlying genetic associations between keratoconus and diabetes mellitus

**DOI:** 10.1016/j.aopr.2021.100005

**Published:** 2021-09-04

**Authors:** Kristin M. Ates, Amy J. Estes, Yutao Liu

**Affiliations:** a Department of Cellular Biology and Anatomy, Medical College of Georgia, Augusta University, Augusta, GA, USA; b Department of Ophthalmology, Medical College of Georgia, Augusta University, Augusta, GA, USA; c James and Jean Culver Vision Discovery Institute, Medical College of Georgia, Augusta University, Augusta, GA, USA; d Center for Biotechnology and Genomic Medicine, Medical College of Georgia, Augusta University, Augusta, GA, USA

**Keywords:** Keratoconus, Genetics, Diabetes, Collagen crosslinking, Oxidative stress

## Abstract

Background Keratoconus (KC) is the most common ectatic corneal disease, characterized by significantly localized thinning of the corneal stroma. Genetic, environmental, hormonal, and metabolic factors contribute to the pathogenesis of KC. Additionally, multiple comorbidities, such as diabetes mellitus, may affect the risk of KC. Main Body Patients with diabetes mellitus (DM) have been reported to have lower risk of developing KC by way of increased endogenous collagen crosslinking in response to chronic hyperglycemia. However, this remains a debated topic as other studies have suggested either a positive association or no association between DM and KC. To gain further insight into the underlying genetic components of these two diseases, we reviewed candidate genes associated with KC and central corneal thickness in the literature. We then explored how these genes may be regulated similarly or differentially under hyperglycemic conditions and the role they play in the systemic complications associated with DM. Conclusion Our comprehensive review of potential genetic factors underlying KC and DM provides a direction for future studies to further determine the genetic etiology of KC and how it is influenced by systemic diseases such as diabetes.

## 1. Introduction

Keratoconus (KC) is a bilateral, progressive ectatic corneal disorder characterized by localized thinning of the corneal stroma and alteration of corneal curvature.^1^ As the cornea adopts a conical shape, this results in myopia, irregular astigmatism, and eventual visual impairment.^1^ The histopathology of KC includes stromal thinning, breaks in Bowman’s layer, focal fibrosis, thickening of the epithelium, and keratocyte apoptosis in the anterior stroma.^2,3^ Although KC is known to be multi-factorial with genetic, metabolic, hormonal, and environmental influences, the exact etiology remains elusive.^4–6^ The risk of developing KC has been associated either positively or negatively with many systemic disorders, including but not limited to, Down syndrome,^7^ connective tissue diseases,^8–10^ autoimmune diseases,^11^ and diabetes mellitus (DM).^9,12–15^ However, the exact etiology of KC and its relationship to systemic diseases, such as DM, remains elusive.

DM may have multiple effects on the cornea, including keratopathy, neuropathy, inflammation, alterations in collagen fibrils, and endothelial cell loss.^16^ Multiple studies have suggested that DM is inversely associated with the risk of KC, suggesting a protective role against the development and/or severity of KC.^13,14,17,18^ This is in contrast to other studies that have reported either 1) a positive association in both prevalence and severity between KC and DM or 2) no significant correlation between the two diseases.^14,19–22^ This discrepancy may be reflective of the varying sample sizes, inclusion/exclusion criteria, sample ascertainment approaches, and populations analyzed (Table 1).

Our goal was to provide a comprehensive review of potential genetic associations between DM and KC. We have outlined various genes implicated in KC risk and summarized their potential roles in DM-induced corneal changes (Table 2). These genes may work through several mechanisms including alterations in corneal biomechanics and collagen crosslinking, alterations in ECM composition and proteolytic activity, as well as increased inflammation and oxidative stress.

## 2. Clinical significance

By gathering information regarding the potential overlapping genetic factors in KC and DM, this review discusses potential therapeutic targets that may slow or halt the progression of KC. In other words, it might be beneficial to manage KC by utilizing the same mechanisms that strengthen the cornea in DM. These mechanisms include but are not limited to shifting the balance in the cornea microenvironment towards increased endogenous collagen crosslinking, decreased extracellular matrix (ECM) remodeling and proteolytic degradation, and decreased inflammation. By regulating local gene expression, this may serve as an alternative form of therapy for those patients in which corneal crosslinking is not an option, such as those with prior history of herpetic keratitis and history of poor epithelial wound healing.^23^ Furthermore, this may reduce the need for corneal transplantation in patients with more severe forms of KC.

## 3. Corneal biomechanics and collagen crosslinking in KC and DM

The cornea is naturally a viscoelastic structure, in that it must be elastic enough to expand into an aspheric half-sphere, but stiff enough to maintain its shape and resist the intraocular pressure (IOP).^24,25^ There is a delicate interplay between the multiple corneal layers and the composition within each layer that contributes to the overall corneal shape. This involves the organization of the collagen structure within each layer, the attachment of proteoglycans and glycosaminoglycans to collagen fibers, the corneal swelling pressure, and the production/degradation of extracellular matrix components.^24^ There are three major mechanisms of collagen crosslinking important in corneal biology, as previously outlined in McKay et al., 2019.^26^ Those include an enzymatic reaction: lysyl oxidase-mediated crosslinking as well as two non-enzymatic reactions: 1) advanced glycation end product (AGE)-mediated crosslinking and 2) photooxidative crosslinking mediated by riboflavin as a treatment option for KC. We will discuss the crosslinking mechanisms in more detail as they relate to the genes involved in KC and DM.

In order to quantify differences in corneal shape and structure, two measurements are commonly obtained: the central corneal thickness (CCT) and corneal hysteresis (CH). While CCT is a gross measure of the overall thickness of the cornea, CH reflects the viscous damping ability of the cornea. CH combines elasticity and viscosity and provides further information on the structural integrity of the cornea. Increased edema in the cornea may give the appearance of stromal thickening and increased CCT. This must be taken into account in systemic diseases such as DM, in which glucose induces increased water retention in multiple tissues, including the cornea.^27,28^

KC is associated with alterations in the corneal biomechanics.^1^ Previous studies have shown altered expression or abnormal localization of extracellular matrix (ECM) components in KC,^29^ with decreased levels of proteoglycan core proteins and abnormal collagen synthesis.^25^ Furthermore, age has been shown to be inversely correlated with the risk of KC^30^ and KC progression,^31^ suggesting that age-related crosslinking is protective against KC and other ectasias.^32^

Conversely, diabetes is known to be an independent cause of endogenous, non-enzymatic crosslinking as high levels of glucose causes glycosylation of corneal fibers and increases AGE-mediated crosslinking in the cornea, causing the cornea to become stiffer.^13,32–34^ As a result, increased CCT and alteration in corneal endothelial cells have been observed in patients with diabetes.^35–39^ Lee et al., 2006 reported that CCT was significantly correlated with the duration of diabetes after controlling for age.^37^ Interestingly, Hager et al., 2009 observed a significant increase in CH in patients with diabetes, although they did not observe a significant increase in CCT after correcting for age, IOP, and gender.^40^ Schler et al., 2012 also reported significantly higher CH and corneal resistance factor (CRF) in patients with poorly-controlled diabetes as compared to healthy subjects and well-controlled diabetes.^41^ Since CH and CRF are correlated to HbA1c, this suggests that the cornea biomechanics could be altered depending on extent of glucose control.^41^ These findings suggest that DM may induce structural alterations in the cornea. In terms of corneal biomechanics, in this section we discussed two genes, *LOX* and *COL5A1*, and their roles in relationship to the corneal biomechanics observed in KC and DM.

### 3.1. LOX

Lysyl oxidase (LOX) is a copper amine oxidase that initiates the physiologic crosslinking of collagens and elastin by oxidizing the side chain of peptidyl lysine, thus generating reactive aldehydes on lysine residues that may cross-react with nearby groups.^26,42,43^ In the eye, the LOX enzyme has been detected in the trabecular meshwork, ciliary body, lens, retina, and cornea.^44–46^ In addition to LOX, there are four LOX-like proteins (LOXL1, LOXL2, LOXL3, and LOXL4) that also catalyze the oxidative deamination of lysine residues in collagen and elastin45.

Multiple genome-wide association studies (GWAS) and candidate association studies have identified the association between sequence variants in *LOX* to both KC risk and CCT, suggesting its potential role in KC pathogenesis.^47–49^ Specifically, a particular SNP rs2956549 in *LOX* has been associated with KC in various ethnicities as well as a meta-analysis.^50–53^ Dudakova et al., 2012 reported altered distribution of LOX expression in the corneal stroma of KC patients, with a reduction in total LOX (both LOX and LOXL) protein activity in corneal fibroblasts derived from KC corneas.^45^ This was consistent with a recent study that reported reduced pre-existing LOX and collagen levels in a patient who developed ectasia after small incision lenticule extraction (SMILE), despite normal preoperative biomechanical evaluation.^54^ Furthermore, ectopic LOX expression in the human corneal fibroblast induced significantly more collagen gel contraction *in vitro,* confirming the role that it plays in strengthening the corneal stroma.^54^

Given LOX’s role in collagen crosslinking, reduced LOX activity in KC may lead to impaired cross-linking which results in corneal ectasia.^45,54^ Although there is a confirmed genetic association of LOXL1 to pseudoexfoliation syndrome and open-angle glaucoma,^46,55,56^ it is still unclear the role that LOXL proteins may play in the cornea. Dudakova et al., 2016 confirmed that LOXL1–4 enzymes were present in all layers of the cornea in cryosection samples and reported lower LOXL2 expression in KC corneas using IHC and Western blot analyses.^57^ Further studies will be required to better understand the expression patterns and activity of LOXL proteins in the cornea.

The exact role of LOX in DM-induced corneal changes remains unclear, as one study failed in observing an increase in LOX-mediated crosslinking in the cornea of DM patients.^33^ However, Chronopoulous et al., 2010 revealed that hyperglycemic conditions increased LOX expression and LOX activity in rat retinal endothelial cells *in vitro* and in diabetic rat retinas *in vivo*.^58^ This is consistent with the finding of increased LOX-dependent crosslinking in skin collagen in patients with diabetes.^59^ Coral et al., 2013 confirmed increased LOX mRNA expression in ARPE-19 cells exposed to high glucose.^60^ Further studies will be required to investigate the effects of DM on LOX activity in the cornea. The increase in LOX activity and expression in response to high glucose in retinal studies suggests that high glucose may have a similar effect in corneal stromal and endothelial cells. Given that the cornea is an avascular structure, there may be differential upstream regulation of *LOX* in response to high glucose concentrations as compared to the retina.

### 3.2. COL5A1

Since collagen is the most abundant protein in the cornea,^26^ it is not surprising that various types of collagen have been implicated in association with CCT and KC, including COL1A1 and COL1A2,^61^ COL8A2,^62^ and COL5A1.^37,63^ The genetic association of COL5A1 with CCT, in particular, reached genome-wide significance after combining data from European, Australian, and Singaporean GWAS.^62–66^ Collagen type V is a regulatory fibril-forming collagen involved in the formation of heterotypic fibrils with collagen type I.^67–70^ Together, both collagen type I and V are the dominant collagen isoforms in the human corneal stroma.^71^ Although collagen type V only comprises 2–5% of the total collagen in most tissues, it determines 10–20% of the fibrillary collagens in the cornea.^67,72^ Interestingly, the most common molecular mechanism in classic Ehler-Danlos syndrome (EDS), a generalized connective tissue disorder, is a functional loss of one COL5A1 allele.^73,74^ Segev et al., 2006 investigated the corneal phenotype in EDS patients with *COL5A1* haploinsufficiency and *Col5a1*^+/−^ mouse models. Both EDS patients and knockout mice exhibited consistent corneal thinning and the *Col5a1*^+/−^ mice also exhibited a decrease in total collagen content with a 25% reduction in the number of stromal fibrils.^72^ These findings suggest that alterations in *COL5A1* expression serves as a strong genetic predisposition towards the development of corneal thinning and KC.

In DM, collagen is influenced by hyperglycemia primarily through nonenzymatic AGE-mediated crosslinking. This occurs by way of the Maillard reaction, in which a primary amine found on amino acid residues, such as lysine or arginine, is converted to a reactive Schiff base that rearranges to form the Amadori product.^26^ This serves as an early-stage product that leads to the formation of AGE products. A classic example of the Maillard reaction is the glycation of hemoglobin, which gives rise to the glycated HbA1c, the established biomarker for sustained glucose levels.^26,75^ Studies have identified increased end products of the Maillard reaction, including pentosidine, within the diabetic cornea.^26,33^ This increase in AGE-mediated crosslinking in DM has been associated with increased tendon stiffness and higher mechanical strength, which could be inhibited by insulin supplementation.^26,76,77^ As a result, the increased crosslinked collagen is resistant to enzymatic degradation in the cornea.^78^ AGE-mediated crosslinked adducts can then bind to the receptor for advanced glycation end products (RAGEs), which are pro-inflammatory receptors expressed in a wide variety of tissues. Although originally described for its ability to bind AGEs, RAGEs have the capability to bind multiple other ligands and play a crucial role in homeostasis and inflammatory processes.^79,80^

To our knowledge no direct associations between *COL5A1* expression and DM have been established. Very recently, Ng et al., 2020 evaluated possible gene-environment interactions between genetic variants identified via GWAS, and the effect of glycemic control (indicated by HbA1c) on the risk of severe diabetic retinopathy (DR).^81^ Interestingly, the SNP *COL5A1* rs59126004 exhibited a protective effect against DR in patients with adequate glycemic control (HbA1c <7%), but not in patients with inadequate glycemic control (HbA1c ≥ 7%),^81^ suggesting a potential interaction between *COL5A1* rs59126004 and glucose levels in the retina.

Priyadarsini et al., 2016 quantified the expression of collagen type I, III, and V in the human corneal stroma of Type 1 and Type 2 DM, and discovered elevated levels of collagen type I and type III, but not collagen type V.^27^ Given that this was a small study with four to eight donor corneas in each category, it would be worth expanding the study with a larger sample size. Collagen type I and V are the predominant types of a healthy cornea, while collagen type III is primarily associated with fibrosis and wound healing in the setting of injury. This finding suggests that hyperglycemia may induce collagen expression in an isoform-dependent manner.^27^ We expect more studies of COL5A1 to better understand DM’s direct effects on *COL5A1* in the cornea.

## 4. Alterations in extracellular matrix (ECM) remodeling in the cornea

In line with collagen crosslinking, alterations in corneal ECM remodeling have been observed in KC and DM including variations in cellular proliferation, disruption in autophagic flux, and increased ECM fibrosis. This is complemented by the observation of an imbalance between matrix metalloproteinase (MMP) and tissue inhibitors of matrix metalloproteinase (TIMPs) in KC patients.^82,83^ Here, we take a close look at several KCand CCT-associated transcription factors including *FOXO1*, *SMAD3*, *TGFBI*, and *ZEB1* (Table 2). We then searched the literature to determine how they may be differentially regulated under hyperglycemic conditions in the cornea. We also included ECM remodeling genes *MMP-9*, *TIMP-1,* and *MIR184*.

### 4.1. FOXO1

Transcription factor Forkhead box protein O1 (FOXO1) is ubiquitously expressed in mammalian cells.^84^ It mediates multiple different pathways, including regulation of metabolic homeostasis, oxidative stress, cell proliferation, and autophagy. Sequence variants near or within *FOXO1* have been associated with CCT,^66,85,86^ but its effect on FOXO1 expression and activity is not well-described in the KC cornea. FOXO1 is regulated by a variety of environmental factors, including insulin, which normally inhibits FOXO1 activity.^87^ In settings of insulin resistance and increased glucose, such as T2DM, increased FOXO1 has been shown to induce gluconeogenesis abnormalities, cell apoptosis, uncontrolled autophagy, and inhibition of proliferation.^84,88^

Although much remains to be understood about FOXO1’s role in KC and DM, some studies have analyzed its effects on vascular endothelial cells. Wilhelm et al., 2016 indicated that FOXO1 decreases the metabolic and proliferative activities of vascular endothelial cells by attenuation of glycolysis and mitochondrial respiration.^84,89^ This study reported that the endothelial-restricted deletion of FOXO1 in mice caused a profound increase in endothelial cell proliferation, such that it induced vascular hyperplasia and vessel enlargement. In contrast, overexpression of FOXO1 resulted in vessel thinning and reduced branching of vasculature.^89^ A separate study reported that FOXO1 signaling was involved in AGE-induced vascular endothelial cell autophagy through impairment of autophagosome-lysosomal fusion. This impaired autophagic flux then resulted in endothelial cell autophagic apoptosis.^90^ These findings are intriguing as they suggest a correlation in FOXO1 activity with AGE-induced endothelial damage and decreased endothelial cell proliferation in the vasculature. Thus, it would be interesting to perform similar studies in the microenvironment of the cornea to determine if differential regulation of FOXO1 may contribute to altered endothelial cell proliferation and/or endothelial cell apoptosis in KC and DM corneas.

### 4.2. SMAD3

There are three functional classes of SMAD proteins, including the receptor-regulated SMAD (R-SMAD), the Co-mediator SMAD (Co-SMAD), and the inhibitory SMAD (I-SMAD) proteins.^91^ SMAD3 is an R-SMAD that is directly phosphorylated and activated by type I receptor kinases, forming activated SMAD complexes that then activate the transcription of target genes,^91^ specifically those involved in the TGFβ signaling pathway. TGFβ signaling is tightly regulated by I-SMAD proteins, SMAD6 and SMAD7, which compete for binding of SMAD3 to co-mediators.^92^ TGFβ signaling is known to play an important role in ECM remodeling and MMP expression.^93–95^

Several studies have identified involvement of TGFβ signaling in KC.^96–99^ Priyadarsini et al., 2015 observed a significant increase in pSMAD3 expression with TGFβ3 signaling in human KC cells compared to normal human corneal fibroblasts.^98^ This was paralleled by significant downregulation of SMAD6/7 at baseline and failure of SMAD6/7 upregulation in response to stimulation with TGFβ in the human KC cells.^98^ Recently, variants near or within *SMAD3* were identified to be associated with KC susceptibility in GWAS of CCT,^49,100^ further reinforcing its potential role in KC pathogenesis. Taken together, it has been suggested that the lack of inhibition by SMAD6/7, along with potential alterations in SMAD3 activity, results in increased TGFβ signaling that may promote the formation of a fibrotic ECM in KC.^98^

TGFβ/SMAD signaling plays an important role in regulating glucose and energy homeostasis as well.^101^ The TGFβ/SMAD3 pathway is activated downstream of the AGE/RAGE signaling pathway in the setting of hyperglycemia.^102,103^ This is suggested to be the main driving force in the development of diabetic nephropathy (DN) due to increased ECM deposition in mesangial cells.^104,105^ Ono et al., 2018 demonstrated that AGE stimulation resulted in significant activation of Smad1 and Smad3 in mesangial cells in mice, likely as a result of increased TGBβ signaling.^106^ Additionally, the loss of Smad3 prevented renal dysfunction under diabetic conditions by reduced mesangial matrix accumulation and reduced GBM thickening.^106^ This reinforces the critical role that TGFβ/SMAD3 plays in ECM remodeling, and shows that this pathway is also affected under hyperglycemic conditions. However, this pathway has not been analyzed in the DM cornea. Interestingly, TGFβ/SMAD3 signaling promoted gluconeogenesis in hepatocyte cells through interaction with FOXO1, another established KC-susceptibility gene.^101^ It is necessary to determine if the TGFβ/SMAD interaction with FOXO1 is present in the human cornea.

### 4.3. TGFBI

The transforming growth factor beta-induced (*TGFBI*) gene has been implicated in the pathogenesis of KC and a heterogeneous group of corneal dystrophies that are characterized by the progressive loss of corneal transparency.^107^ It encodes transforming growth factor beta-induced protein (TGFBIp), which is also known as keratoepithelin, BIGH3, or βigh3.^108^ In the cornea, it is expressed primarily in the corneal epithelium, stroma, and retrocorneal fibrous membranes.^108–112^ TGFBIp is known to interact with multiple extracellular macromolecules, including the proteoglycan decorin^113^ and collagen type I, II, IV, VI, and XII.^114–117^ It is thought to link cells to the ECM through various integrin binding sites^116,118–122^ and thus play important roles in corneal wound healing and maintenance of the ECM.

In a cDNA library constructed from KC corneas, *TGFBI* was found to be the second most abundant transcript.^123^ Sequence variants in *TGFBI* were also identified in Chinese and Polish KC patients.^124,125^ However, the exact relationship between *TGFBI*, TGFBIp, and KC is still unclear. Researchers have suggested that mutations in *TGFBI* could contribute to decreased mechanical stability in the cornea, thus resulting in corneal thinning as seen in KC.^126^ This is supported by findings of decreased TGFBIp in KC corneas.^126^ Conversely, elevated levels of TGFBIp have been found in areas of corneal scarring, likely due to TGFB1-mediated upregulation in response to corneal injury.^126–128^ Interestingly, two KC patients had stromal amyloid deposits that were associated with TGFBIp in the corneal buttons, but no *TFGBI* mutation was present.^99^ It is possible that there was concurrent scarring in these two patients, or that local factors in the KC corneas predispose to development of TGFBIp amyloid deposits, thus disrupting the structural integrity of the cornea.^99^

*TGFBI* has also been identified as a potential risk gene for the development of both T1DM and T2DM after detecting the association of several SNPs in human genetic studies.^129^ Aside from the cornea, TGFBIp is produced by smooth muscle cells, fibroblasts, and proximal tubular epithelial cells^130,131^ upon TGFβ or high-glucose stimulation.^127,132^ In studies investigating DM, it was observed that high glucose levels increased TGFBIp expression in renal proximal tubule epithelial cells by activating TGFβ. This coincided with findings of a high glucose-stimulated increase in collagen and fibronectin production in mesangial cells and proximal tubular cells, which is mediated by TGFβ activation.^133–135^ Although the pathologic consequences of increased TGFBIp in the proximal tubules remains unclear, it may play an important role in the degree of renal interstitial fibrosis, which is closely correlated with a progressive decline in renal function in DM.^132,136,137^ To date, no studies have analyzed *TGFBI* and TGFBIp in the diabetic cornea, but TGFBI has been shown to be upregulated *in vitro* in the human corneal epithelial cell line in response to TGFβ.^128^ Given these findings, it is possible that TGFBIp may also be increased in the cornea in response to high glucose, promoting TGFβ-mediated corneal thickening and fibrosis. Additionally, it has been suggested that TGFBIp mutations occur in a genotype-phenotype fashion, in which various mutations account for the different degrees of phenotypic severity seen in KC and corneal dystrophies.^138^ Thus, the high glucose environment seen in DM could result in a variation on the phenotypic spectrum of *TGFBI* mutations and/or epigenetic modifications.

### 4.4. ZEB1

The zinc finger E box-binding homeobox 1 (*ZEB1*), also known as transcription factor 8 (*TCF8*)^139,140^ or δEF1,^141^ can function as either a transcriptional enhancer or repressor for different genes.^142^ ZEB1 has been implicated in the regulation of type 1 collagen expression, particularly in osteoblasts,^141^ and repression of the epithelial phenotype.^140,143,144^ It has been identified as a potent epithelial to mesenchymal transition (EMT) activator and stimulator of angiogenesis in tumor biology studies,^145–147^ as well as a regulator of TGFβ signaling with its counterpart, ZEB2.^148^

Mutations in *ZEB1* have been reported in posterior polymorphous corneal dystrophy (PPCD),^139,140,149^ Fuch’s endothelial corneal dystrophy (FECD),^127,150,151^ and keratoconus.^152^ This highlights another example of genetic heterogeneity that results in a variety of phenotypic presentations, like that of *TGFBI*. A heterozygous frameshift mutation in *ZEB1* was found to induce ectopic expression of *COL4A3* by corneal epithelial cells in PPCD, implicating *COL4A3* as a possible target of *ZEB1* regulation.^140^ In contrast, a missense *ZEB1* mutation resulted in markedly reduced *COL4A1*, *COL4A2*, and *COL4A3* expression in corneal keratocytes.^152^ Reduced expression of *COL4A1* and *COL4A3* has also been reported in KC,^29,153^ but further work is required to determine the role of *COL4A1*/*COL4A3* and how they may be regulated by *ZEB1* in KC. Based on current findings, Lechner et al., 2013 suggested that missense mutations in *ZEB1* are associated with FECD and KC, while protein truncating mutations result in PPCD.^152^ This is supported by the finding of unique mutations in a family with both KC and FECD, as well as a patient with triple corneal dystrophy consisting of KC, FECD, and epithelial basement membrane corneal dystrophy.^152,154^

In efforts to better understand ZEB1’s role in wound healing and angiogenesis, it was recently found that persistent hyperglycemia, as seen in DM, potently induced *ZEB1* expression in human dermal microvascular endothelial cells (HMEC).^155^ This corresponded with increased *ZEB1* expression in laser capture microdissection endothelial tissue obtained from the wounded edge of diabetic wound patients.^155^ In Singh et al., 2019, immunoprecipitation-mass spectrometry was performed to gain further mechanistic insights into the differential action of ZEB1 under normoglycemic and hyperglycemic conditions, thus revealing putative proteins that physically associated with ZEB1.^155^ It was found that hyperglycemia diminished the physical association of ZEB1 with E-cadherin, resulting in a loss of control over E-cadherin repression which is known to cause the microvascular endothelial dysfunction commonly observed in DM.^155,156^ Additionally, hyperglycemic conditions impaired the binding of several pro-inflammatory proteins, suggesting that alterations in cellular ZEB1 may contribute to the inflammation seen in DM.^155^

A different study analyzed the role of long noncoding RNA (lncRNA) ZEB1 antisense 1 (*ZEB1-AS1*) in DM, as it has been shown to increase ZEB1 expression.^157,158^ By increasing ZEB1 expression, ZEB1-AS1 is thought to play an antifibrotic role in DM through modulation of EMT, which is considered to be the main pathogenic factor of renal fibrosis.^158–160^ Wang et al., 2018 confirmed this in ZEB1-AS1 knockdown studies, which increased high glucose-induced ECM accumulation by downregulation of ZEB1 expression, resulting in renal fibrosis.^161^ This was investigated further by Meng et al., 2020, who reported that *ZEB1-AS1* was down-regulated in kidney tissues of DM patients as well as hyperglycemic-induced HK-2 cells.^158^ Furthermore, ZEB1-AS1 improved the high glucose-induced EMT and fibrogenesis by mediating miR-216a-5p and BM7.^158^ While the exact mechanisms are beyond the scope of this review, it is clear that there is a very complex mechanism of regulation surrounding ZEB1. It is important to study the role of *ZEB1* in the cornea of DM patients as it may have the potential to alter the expression of inflammatory cytokines and disrupt corneal endothelial/epithelial structure under hyperglycemic conditions.

### 4.5. MMP-9 and TIMP-1

MMPs are a family of 24 zinc-dependent proteases involved in multiple physiological processes including tissue remodeling and the degradation of ECM.^162,163^ The activity of the MMPs is balanced by four inhibitory proteins, the tissue inhibitors of metalloproteinases (TIMPs1–4).^164,165^ Multiple MMPs have been implicated in the pathogenesis of KC, including MMP-1, MMP-2, MMP-3, MMP-7, and MMP-13.^166,167^ MMP-9, also known as gelatinase B, is among the most well-studied in relation to KC.^164^ Multiple studies of tear composition have shown increased levels of MMP-9 protein in KC, including one patient with asymmetrical KC in which MMP-9 was upregulated only in the tears of the affected eye.^168–170^This increase in MMP-9 was confirmed with an accompanying upregulation of *MMP-9* mRNA in the corneal epithelium.^170^ Interestingly, the *MMP-9* mRNA in KC patients was significantly higher in cells from the cone apex as compared to the corneal periphery, which may contribute to the focal structural weakness of the cornea.^171^ An additional study revealed an increase in MMP-9 protein level in the blood of KC patients compared to controls.^172^

TIMP-1 exhibits a unique binding interaction with MMP-9, as it usually exhibits a high level of coordinated expression with MMP-9, is frequently secreted as a TIMP-1/MMP-9 complex, and binds MMP-9 with high affinity.^173^ Recent studies revealed a decrease in the levels of TIMP-1 in KC corneas.^83,174,175^ Taken together, the increased MMP-9 and decreased TIMP-1 activity seen in KC may reflect in an imbalance of proteolytic activity, thus contributing to ECM degradation and corneal thinning. This is reinforced by the analyses of genetic polymorphisms in *MMP-9* and *TIMP-1* in KC patients that were associated with findings of higher MMP-9 and lower TIMP-1 activity in KC tear samples.^176^ It is important to note that the *TIMP-1* SNP was only associated with increased KC risk in females.^177^ Given that *TIMP-1* is located on the X chromosome,^178^ this likely accounts for differences in gender susceptibility.

In regards to DM, studies have indicated that elevated glucose levels also disrupt the MMP/TIMP balance in macrophages and endothelial cells, primarily through an amplification in MMP expression and activity.^179^ Takahashi et al., 2000 reported enhanced MMP activity in human corneal epithelial cells under hyperglycemic conditions as well as increased MMP-9 activity in the cornea of diabetic rat models.^180^ In support of this finding, two studies reported: 1) increased MMP-9 and TIMP-1 protein levels in the tears of pediatric T1DM patients along with 2) increased MMP-9 activity in tears of T2DM patients.^181,182^ Additionally, genetic polymorphisms in *MMP-9* have been associated with susceptibility to T2DM and diabetic nephropathy.^183–185^ However, no genetic association has been identified with *TIMP-1* and DM in patients.^185^ The role of TIMP-1 in DM remains inconclusive. Taken together, differences in the imbalance of MMP-9/TIMP-1 activity may contribute to different degrees of ECM remodeling in the cornea of KC and DM patients.

### 4.6. MIR184

MicroRNAs (miRNAs) bind to the complementary sequences located in the 30 -UTR region of the target genes, resulting in degradation of the mRNA or suppression of translation.^186^ *MIR184*, in particular, encodes for miR-184, which is expressed in the central corneal epithelial cells and the lens epithelium.^187–189^ It is the most abundant miRNA in both the cornea and the lens and it is known to competitively inhibit the binding of miR-205 to the mRNA of the inositol polyphosphate phosphatase-like 1 gene (*INPPL1*, also known as *SHIP2*).^189,190^ This neutralizes the inhibitory activity of miR-205 on INPPL-1 and subsequently downregulates the Akt pathway, which has been shown to markedly increase keratinocyte apoptosis and cell death.^190^ Interestingly, this situation is unique to the corneal epithelium as it is the only known epithelium that exhibits overlapping expression of miR-184 and miR-205.^189,190^ Additionally, miR-184 has been shown to regulate differentiation of human-induced pluripotent stem cells into corneal epithelial-like cells.^191,192^

Given its role in the cornea, it is not surprising that mutations in *MIR184* may lead to KC. To date, multiple studies have identified a heterozygous mutation in *MIR184* in a Northern Ireland family with KC and cataracts,^187^ a family with EDICT (endothelial dystrophy, iris hypoplasia, congenital cataract, and stromal thinning) syndrome,^193^ and an European Spanish family with various corneal abnormalities including severe KC.^194^ However, the lack of mutations in KC patients from Iran,^191^ Turkey,^195^ and Saudi Arabia^196^ suggests a limited role of *MIR184* in KC pathogenesis.^196^

Specific miRNAs have also been associated with T2DM cellular processes, including apoptosis, response to cytokines, and insulin secretion.^197^ miR-184, in particular, has been identified as an important modulator of compensatory pancreatic β-cell expansion during insulin resistance.^198–200^ Generally, the expression of miR-184 is increased in islet cells in periods of fasting, demonstrating an active role in pancreatic β-cells as the glucose levels decrease.^201^ Likewise, the miR-184 expression levels have been shown to decrease in the presence of increasing extracellular glucose.^201^ Together, this highlights the potential role of miR-184 in glucose metabolism. Furthermore, its expression is strongly decreased in the pancreatic islet cells of insulin-resistant mouse models and human patients with T2DM.^200^ miR-184 expression and activity needs to be further studied in the DM cornea. Given its regulation by glucose levels in pancreatic islet cells, it is possible that miR-184 may be similarly downregulated by high glucose levels in the DM cornea, thus inhibiting cellular apoptosis and resulting in corneal thickening.

## 5. Inflammation and oxidative stress in the cornea

Although KC has traditionally been described as a noninflammatory degenerative condition, there is emerging evidence suggesting that inflammation within the epithelium and stroma are involved in the pathogenesis of KC.^3^ Multiple studies have observed significant increases in proinflammatory molecules such as IL-6, IL-4, IL-5, IL-8, and IL-12^169,202^ in KC tears compared to controls. Additionally, KC keratocytes have been reported to express more IL-1α receptors,^203^ which may trigger keratocyte apoptosis since IL-1α is a proinflammatory cytokine.^204^

In parallel, several studies have found that oxidative stress is involved in the development and progression of KC.^83,205–207^ KC corneas have exhibited abnormalities such as increased levels of inducible nitric oxide synthase, nitrotyrosine, malondialdehyde, and glutathionine S-transferase,^208^ as well as decreased activities of extracellular superoxide dismutase.^205^ This is supported by multiple observations of KC corneas and fibroblasts exhibiting increased levels of ROS and relatively greater mitochondrial DNA damage as compared to controls.^207,209,210^ Interestingly, the combination of oxidative stress and hyperglycemia, particularly in T2DM, accelerates AGE formation.^80^ Thus, with increased AGE accumulation, this may create the potential for increased inflammation, enhanced production of ROS, and impairment of DNA repair mechanisms^80^ in the DM cornea as well.

The increase in inflammatory markers and ROS within the KC cornea may be the result of environmental stimuli such as eye-rubbing and external oxidants such as UV light, which drives the pathological thinning of the stroma.^211^ DM itself is an inflammatory systemic disease, so we wanted to explore its effects on the KC-associated genes. In this section, we focused on *HGF*, *CAST*, *SOD1*, and *IL1A/IL1B* and their roles in DM and KC pathophysiology (Table 2).

### 5.1. HGF

Hepatocyte growth factor (HGF) is a pleiotropic growth factor that binds to its receptor, mesenchymal-epithelial transition factor (c-Met/ Met) and activates multiple downstream pathways, including MAPK, PI3K-Akt axis, and activators of transcription (JAK/STAT) pathways.^212^ It is primarily involved in cell proliferation and migration, particularly in corneal epithelial cells, as well as inflammatory-related signaling cascades.^3,213,214^ Injured corneas have exhibited increased *HGF* and *c-Met* mRNA expression during corneal wound healing.^214–216^

The genetic association between *HGF* variants and KC susceptibility was identified with several *HGF* SNPs in the European and Australian cohort studies.^50,217,218^ Given that all the currently identified SNPs are located in a noncoding region upstream of *HGF*, it was suggested that they regulate gene expression by way of RNA splicing, transcription factor binding, and miRNA regulation.^3,219^ Additionally, there is recent evidence of increased HGF protein in the KC epithelium compared to control corneal epithelium.^3^ This suggests that poorly regulated and overexpressed HGF may have detrimental effects on the ECM due to inflammation in KC.

In diabetic corneas, both *ex vivo* and organ culture, HGF expression was noted to be increased with a corresponding decrease in HGF receptor c-Met expression.^220^ This suggests a disruption in the HGF/c-Met system, such that there is reduced cell migration and poor epithelial healing, which is characteristic of diabetic corneas.^221–223^ This hypothesis is supported by the finding that overexpression of c-met in diabetic corneas resulted in restoration of nearly normal epithelial wound healing times.^223^ In other DM studies, HGF has been shown to play a role in the metabolic flux of glucose, manage β-cell homeostasis, and modulate the inflammatory response.^224^ Thus, it is possible that reduced receptor c-Met expression in DM corneas could serve a protective role against the inflammatory response mediated by HGF.

### 5.2. CAST

*CAST* encodes calpastatin, an inhibitor of calpains. CAST is a calcium-dependent cysteine protease that is involved in a variety of cellular processes, including proliferation, apoptosis, and cell migration.^225^ The calpain/calpastatin system is present in the corneal epithelium where it is suspected to play a role in epithelial cell turnover and wound healing.^226,227^ It has also been localized to corneal endothelial cells and fibroblasts.^226,228^ Genotyping of both Caucasian and Han Chinese patients with KC revealed a consistent association between variants near or within *CAST*, with KC susceptibility.^226,229^

In DM, calpain activity has been shown to be increased in vascular endothelial cells in response to excess glucose.^127,230–233^ Calpain may also play a role in mitochondrial ROS generation, such that it contributes to diabetic vascular injury by way of vascular inflammation^232,234^ and glucose-induced apoptosis in endothelial cells.^230,235^ This is supported by the finding that genetic inhibition of calpain through over-expression of calpastatin reduces vascular ROS production.^230^ Increased glucose-induced calpain activity has also been shown to initiate vascular endothelial dysfunction by inactivating prostacyclin (PGI_2_), as overexpression of endogenous calpastatin inhibited this effect.^236^ A similar mechanism may be observed in DM corneas, as calpain is activated by high-glucose levels. Analyzing the expression and activity levels of CAST in KC and DM corneas will give us further insight into this pathway.

### 5.3. SOD1

Superoxide dismutase 1 (*SOD1*) encodes a copper and zinc-dependent cytoplasmic enzyme that is directly involved in the antioxidative processes associated with ROS elimination and reduction of oxidative stress in the cornea.^205^ It has been shown to localize to the corneal epithelium, endothelium, and keratocytes.^205^ Given that there have been increased levels of oxidative stress markers in the KC cornea, several studies have suggested that mutations in *SOD1* might be involved in development of KC.^48,83,237^ A specific *SOD1* deletion was detected in two non-related American families with an autosomal dominant form of KC as well as in the Greek and Brazilian KC population.^238–240^ Mutational analyses revealed that this deletion excluded the SOD1 protein active site, which suggests a loss of enzyme function.^239^ Although the specific SOD1 deletion has had a low frequency in cohort studies, the fact that it has been identified in various populations suggests that it might serve as a potential genetic component in the development of KC. This is supported by the finding of suppressed levels of *SOD1* expression in KC corneal fibroblast cultures as compared to controls.^241^ However, this is a controversial topic as other studies have refuted SOD1’s involvement as there was not enough evidence in mutational analyses.^48,127,237,242–246^

Mutations in the *SOD1* gene has also long been associated with amyotrophic lateral sclerosis (ALS). It is generally accepted that mutations in *SOD1* results in conformational instability of the protein, resulting in the formation of SOD1 aggregates that exert a cytotoxic effect in motor neurons, which then results in the progressive degeneration of motor neurons observed in ALS.^247^ Interestingly, ALS has recently been shown to share several genetic pathways with DM.^248^ Additionally, several genetic variations of *SOD1* polymorphism have been associated with diabetes and diabetic complications.^249–254^ One study implicated a role for AGE/RAGE signaling in DM-mediated vascular calcification through activation of Nox-1 and decreased expression of SOD1, which increased oxidative stress.^255^ Recently, a separate study investigated ocular surface damage in diabetic mice and found an accumulation of ROS, increased expression of RAGE, and decreased *SOD1* expression in the cornea.^256^ This study also reported that topical treatment with pigment epithelium-derived factor (PEDF) was shown to improve corneal epithelial damage by decreasing RAGE and increasing SOD1 expression.^256^ Together, this suggests that SOD1 may play an important role in alleviating the oxidative stress seen in corneal pathology. SOD1’s role in KC pathogenesis is currently under debate, but it is possible that increased AGE/RAGE activity in the DM cornea results in decreased SOD1 expression.

### 5.4. IL1A and IL1B

Interleukin (IL)-1 is a proinflammatory cytokine involved in various cellular activities, including cell proliferation, differentiation, and apoptosis.^257^ The IL-1 family includes two proinflammatory cytokines, IL-1α and IL-1β, and the IL-1 receptor antagonist (IL-1Ra), encoded by *IL1A*, *IL1B*, and *IL1RN*, respectively.^257^ IL-1 has been shown to upregulate keratocyte expression of collagenases, metalloproteinases, and other enzymes involved in collagen remodeling during corneal wound healing.^258,259^ Early studies detected increased keratocyte apoptosis in KC corneas and suggested that it might be triggered by increased basal IL-1 release.^260,261^ This is supported by the finding of increased IL-1α binding sites in KC corneal fibroblasts compared to control corneas.^203^ However, genetic association analyses for polymorphisms in *IL1A* and *IL1B* in KC have remained controversial. Genetic associations with KC have been identified with variants in *IL1B* in the Han Chinese,^262^ Korean,^263^ and Japanese KC population,^257^ but the involvement of *IL1A* was only observed in the Han Chinese KC population.^262^ Furthermore, no genetic associations with variants in or near *IL1A* or *IL1B* were observed in the Turkish population.^264^ To date, it is still unclear how the mRNA expression levels of *IL1A* and *IL1B* relates to the expression of cytokines IL-1α and IL-1β in KC pathophysiology.

The IL-1 family of cytokines has also been implicated in DM and DM-related complications by way of inflammation.^265–268^ To better understand the role of IL-1 in the diabetic cornea and corneal wound healing, a recent study utilized a genome-wide cDNA array analysis in normal and DM mouse corneas.^268^ This study by Yan et al., 2016 reported upregulation of IL-1β expression in the healing corneal epithelium of both normal and DM corneas, with no difference in IL-1α expression.^268^ There was a corresponding increase in *IL1RN* expression in the normal cornea, but a decrease in the DM cornea, suggesting a disturbed balance of IL-1β to IL-1Ra.^268^ This disruption in IL-1Ra signaling had multiple adverse effects in corneal wound healing, including suppression of proinflammatory cytokine/chemokine expression and a decrease in the overall early inflammatory response to wounding in DM mouse corneas.^268^ Interestingly, increased production of IL-1β has also been observed in macrophages in response to prolactin stimulation.^269^ Given that prolactin-induced protein has recently been suggested to be a novel biomarker for KC,^270,271^ this is an intriguing association between the balance of IL-1β to IL-1Ra signaling and hormonal influences.

A separate cross-sectional analysis reported increased levels of IL-1Ra in patients with prevalent DM or metabolic syndrome.^162^ It was suggested that the increased IL-1Ra expression levels predicted the progression of metabolic syndrome to clinically incident diabetes, independently of CRP and other risk factors.^162^ This study also revealed genetic variants in *IL1A*, *IL1B*, and *IL1RN* that may have gender-specific associations with DM.^162^ Further studies will be required to better understand how the genetic variants in *IL1A* and *IL1B* influence expression of IL-1α and IL-1β and whether there is differential regulation of the IL-1 family in KC and DM.

## 6. Additional genes of interest

Multiple other genes have been associated with CCT and KC pathophysiology, including but not limited to *SPRY2* and *COL4A3*/ *COL4A4*^48,49^ (Table 2). Here, we discuss how these genes may potentially have overlapping or differing roles in KC and DM pathogenesis.

### 6.1. SPRY2

The Sprouty family consists of four members, SPRY1–4. All are direct targets and negative feedback regulators of fibroblast growth factor (FGF) signaling,^272–274^ playing important roles in the early development of multiple organs.^273,275,276^ SPRY2, in particular, has been shown to modulate the apoptotic actions induced by pro-inflammatory cytokine, TNF-α.^277^ Kuchara et al., 2011 revealed an important role for Spry1 and Spry2 in the regulation of lens vesicle separation and corneal epithelial proliferation in mouse models.^278^ This study generated *Spry1;Spry2* double-null mutants and observed increased corneal epithelial proliferation and an inhibition in terminal differentiation.^278^ A later study revealed that eyelid closure was impaired due to increased proliferation of conjunctival epithelial cells in Spry conditional knock-out mutants.^279^ This suggests that Spry-1 and Spry-2 normally suppress ectopic growth in the corneal epithelial tissue. A recent GWAS identified *SPRY2* as a novel candidate gene significantly associated with CCT inter-individual variation.^49^ Although no studies have analyzed SPRY2 in KC pathogenesis, it is possible that it may contribute to alterations in the corneal epithelium.

Recently, a variant near *SPRY2* was found to be associated with increased susceptibility to T2DM in both the Han Chinese and Japanese population.^280,281^ *SPRY2* KO in human hepatocyte cells resulted in increased glucose intake, suggesting a possible role for SPRY2 in glucose metabolism in hepatocytes.^282^ This same study also reported the upregulated genes in *SPRY2* KO cells to be involved with DNA replication and cell cycle regulation, which is consistent with its established role in inhibition of cellular proliferation.^282^ Taken together, deletions in *SPRY2* may have pathologic effects in both the cornea and metabolic homeostasis.

### 6.2. COL4A3/COL4A4

*COL4A3* and *COL4A4* both encode for collagen type IV, the major structural component in epithelial and endothelial basement membranes in the human cornea.^283,284^ Collagen type IV mutations have been implicated in a variety of clinical manifestations, including Alport syndrome and PPCD.^140,283,285^ Both genes have been reported to be differentially expressed in KC corneas, suggesting a role for collagen type IV in KC pathogenesis.^153,286^ However, in genetic analyses of *COL4A3* and *COL4A4*, no pathogenic variants were identified in KC patients, although common polymorphisms are present in the affected and healthy populations.^48,86,283,287,288^ Thus, the role of collagen type IV mutations in KC pathogenesis is unclear.

Collagen type IV is also a major structural component of the glomerular basement membrane (GBM), and alterations in the collagen composition have been implicated in the pathogenesis of nephropathy in DM.^289^ GWAS meta-analysis of T1DM revealed a SNP rs55703767 that resulted in thinner GBM in patients with DM but was protective against renal complications.^290^ Interestingly, Onochie et al., 2020 reported that hypoxia induced a reduction in laminin and collagen type IV in the cornea, which resulted in a delay in wound healing and increased corneal stiffness.^291^ This was in parallel to the finding of impaired wound healing and a decrease in laminin along the basal lamina in the diabetic cornea.^284^ Given that diabetes can cause a transition to a hypoxic state, this suggests a potential role for collagen type IV in the induction of increased corneal stiffness that has been reported in DM.^291^ Further studies will be needed to see if collagen type IV mutations have a potential effect on the DM cornea and to better understand how hypoxia affects collagen type IV.

## 7. Discussion

Multiple retrospective epidemiological studies have suggested that patients with DM have reduced risk in the development and/or severity of keratoconus (Table 1).^13,14,17,18^ Seiler et al., 2000 was among the first to report this protective role of DM in a retrospective case-control study of the German population.^13^ This finding was later confirmed by Naderan et al., 2014 in the Iranian population,^17^ and Kuo et al., 2006 in the United States population.^14^ One limitation of the above studies is that the data was extracted from the hospital/clinic population. Woodward et al., 2016 addressed this by conducting a retrospective longitudinal population-based cohort study in the United States.^18^ With a larger sample size, Woodward et al., 2016 found that there were 20% lower odds of KC development with uncomplicated DM and 52% lower odds of KC development with DM-associated complications.^18^

**Fig. 1.**
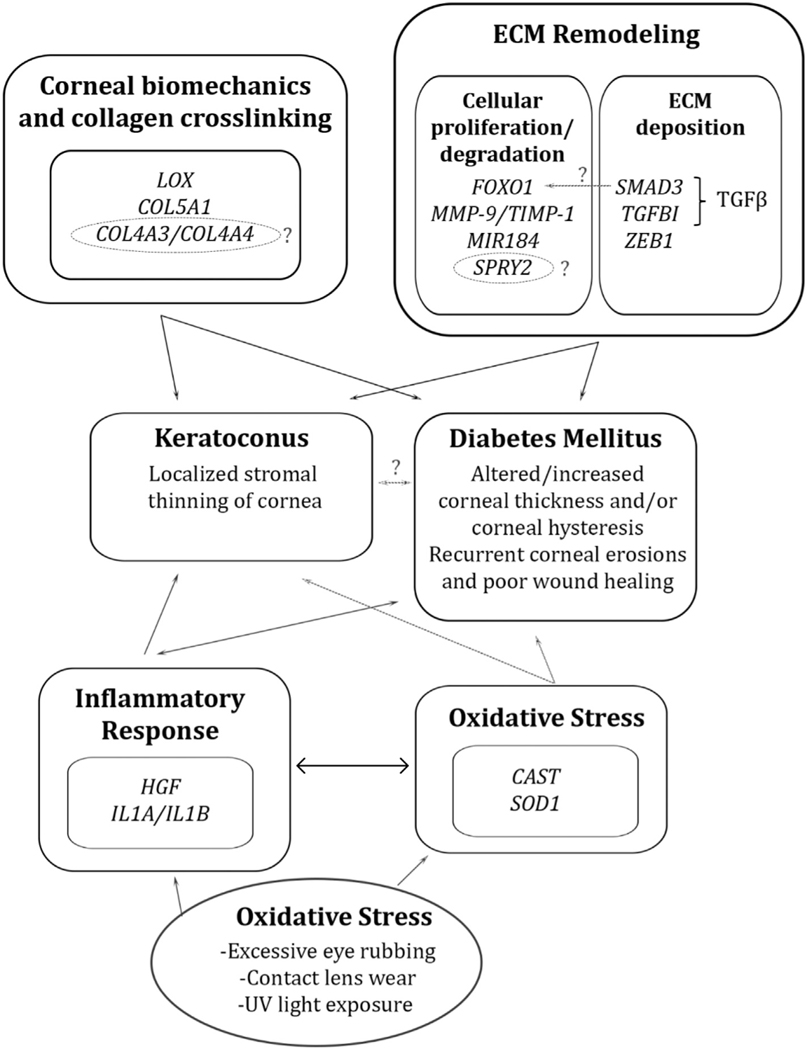
In KC and DM, several overlapping mechanisms may contribute to the discussed pathology, including alterations in corneal biomechanics and collagen crosslinking, alterations in ECM composition and proteolytic activity, as well as increased inflammation and oxidative stress.

A common working hypothesis in support of this observation is that chronic hyperglycemia promotes glycosylation of corneal fibers and induces endogenous collagen crosslinking within the corneal stroma, thus preventing the biomechanical weakening of the cornea.^13,34^ Although the cornea is an avascular structure, previous reports have indicated that corneas in diabetic patients are still exposed to increased glucose concentration,^292–294^ which supports this hypothesis. Another working hypothesis is that DM disrupts corneal endothelial cell function, resulting in stromal edema and increased CCT^35,37,295–297^ (Fig. 1).

This remains a debated topic as other studies have either 1) suggested a positive association between KC and DM or 2) did not identify a significant correlation (Table 1).^14,19–22^ For example, Kosker et al., 2014 conducted a retrospective case-control and cross-sectional study in the United States population and reported a higher prevalence of T2DM in the KC population as well as greater severity of KC in DM patients.^20^ However, this was clinic-based, and the prevalence of DM in clinic populations as compared to the general population may account for differences in these findings. Subsequently, Moon et al., 2020 conducted a retrospective cohort population-based study in the Korean population and they also reported a higher prevalence of T2DM in the KC population with a positive association of KC and DM.^21^ In contrast, Bak-Nielsen et al., 2019^19^ and Lee et al., 2020^298^ conducted a retrospective cohort study in the Danish and Korean population, respectively, and found no significant differences in DM prevalence in KC patients. In efforts to consolidate the findings, Hashemi et al., 2020^299^ conducted a meta-analysis and reported that although the odds of developing KC were 23% lower, this relationship was not significant. A limitation of this meta-analysis is that all studies included were either cross-sectional or case-control studies, which can be more prone to information and selection bias compared to cohort studies.^299^ Thus, the variations in study designs likely contribute to differences in findings.

The multiple effects of DM on the cornea are complex and have been reviewed elsewhere.^16,26,300,301^ These DM effects include, but are not limited to, keratopathy, neuropathy, inflammation, alterations in collagen fibrils, endothelial cell loss, and increased glucose in the aqueous humor.^16,292^ Patients with DM are often predisposed to ocular surface complications such as dry eye, recurrent corneal erosions, and bacterial infection.^26,302–304^ However, the mechanism of correlation between DM and KC pathogenesis remains unclear. In efforts to better understand KC pathophysiology, multiple candidate and genome-wide association studies have identified the genetic and metabolic components involved in KC.^48,49,100,305^ Additionally, a recent review outlined important biological and chemical pathways related to collagen crosslinking in DM and KC.^26^

In terms of corneal biomechanics, KC is characterized by localized stromal corneal thinning and reduced CCT,^1^ while DM generally causes the cornea to become stiffer with increased CCT and/or CH.^13,34,35,37^ This is likely reflected by changes in the collagen composition of the cornea, as outlined by McKay et al., 2019.^26^ Two genes, *LOX* and *COL5A1*, may play important roles in corneal collagen crosslinking. Current evidence suggests that overall LOX activity (including LOX and LOXL proteins) is decreased in KC, leading to impairment of lysyl oxidase-mediated crosslinking and weakening of the cornea.^45,54^ In contrast, hyperglycemic conditions have been shown to upregulate LOX expression and activity in retinal cells.^58,60^ If a similar effect may be seen in corneal cells under hyperglycemic conditions, this could explain the reduced risk of KC development in DM patients. Given that *COL5A1* reached genome-wide significance in KC patients, and that patients with *COL5A1* haploinsufficiency exhibited corneal thinning, we discussed whether *COL5A1* shared similar genetic significance in DM corneal pathology. To our knowledge, no studies have analyzed DM’s direct effects on *COL5A1* in the cornea. A recent study did find differential collagen composition with elevated levels of collagen types I and type III, but not collagen type V, in the human corneal stroma of DM patients. This finding suggests that hyperglycemia may induce collagen modulation in an isoform-dependent manner. Further studies are needed to better understand the effects of hyperglycemia and increased AGE-mediated crosslinking on the various collagen isoforms in DM corneas, and to determine whether sequence variants in or near *COL5A1* are associated with KC and DM cornea pathology.

Alterations in corneal biomechanics is also characterized by disruption in the ECM composition of the cornea. Thus, we explored several genes that play a role in ECM remodeling and were identified in KC and CCT-associated GWAS. This includes several transcription factors including *FOXO1*, *SMAD3*, *TGFBI*, and *ZEB1.* It became readily apparent that the investigation of these genes is lacking in the DM cornea, but many exhibited an association with AGE-mediated signaling and differential expression under hyperglycemic conditions in other tissues. This suggests that a similar effect could be seen in the DM cornea, but further research studies will be needed to confirm this. TGFβ signaling would be worth exploring further in the KC and DM cornea, as it has been associated with alterations in SMAD3, TGFBIp and FOXO1 signaling. For example, increased TGFβ signaling and SMAD3 activity has been suggested to increase ECM deposition in mesangial cells, leading to the development of DN. This, in combination with an imbalance in MMP-9/ TIMP-1 activity, may contribute to different degrees of ECM remodeling in the cornea of KC and DM patients. Additionally, *MIR1*84 has a very unique role in the remodeling of the corneal epithelium. Given its downregulation by high glucose levels in pancreatic islet cells, *MIR184* may potentially link elevated glucose levels and alterations in corneal epithelial cell apoptosis in the DM cornea.

Interestingly, there was genetic overlap in several inflammatory proteins and regulators of oxidative stress in both KC and DM. Although KC was previously described as a noninflammatory condition, it has been suggested that external environmental factors such as excessive eye rubbing may induce an increase in inflammatory markers, contributing to KC pathogenesis. Likewise, DM has been associated with low-grade inflammation that is thought to contribute to insulin resistance observed in T2DM.^306^ In this review, we closely examined the role of *HGF*, *CAST*, *SOD1*, and *IL1A/IL1B*. While *HGF*’s role remains unclear, the imbalance in HGF and HGF receptor c-Met expression in DM corneas is intriguing, which could lend towards a protective role against inflammatory degradation in the ECM of DM. We found that *SOD1* likely plays a similar role in reducing oxidative stress, as decreased SOD1 expression has been suggested in both KC and DM. Further studies into the calpain/calpastatin system, as well as the ratio of IL-1α/IL-1β to IL-1Ra, will be needed before an association can be made between DM and KC. It is possible that imbalances in both systems may contribute to the differing clinical pathology observed in the cornea.

Last but not least, multiple other genes have been associated with KC pathophysiology, but their roles in DM are unclear. We highlighted a few remaining KC candidate genes, including *SRPY2* and *COL4A3/COL4A4*. However, much more extensive studies are required before correlations can be made with DM. We suggest exclusively studying the DM cornea with a focus on KC-associated genes and analyzing the expression patterns of associated proteins.

From a clinical perspective, the age of onset for KC often ranges from the teenage years to the 30s and 40s while the age of onset for type 2 DM ranges from teenage years to the 70s.^1,271,307–309^ Type 2 DM is becoming increasingly prevalent in children and adolescents worldwide, including the US. The average age of diagnosis for children and adolescents in the US is 14 years.^307^ The incomplete overlap in age-onset between KC and DM may complicate efforts in interpreting the potential protective effect of DM on KC. This raises an important question to all the reported studies: what is the distribution of DM age-onset for those with KC or without KC? It will be more beneficial to only include DM patients with age-onset less than 30–40 years.

## 8. Conclusion

Despite the conflicting literature evidence between KC and DM, our comprehensive discussion explained whether and how DM is associated with KC and how DM may function as a protective mechanism (Fig. 1). At this time, there is more evidence to support the protective role of DM in KC patients. If true, we hope to identify potential localized therapeutic targets in KC management that act by strengthening the KC cornea through similar mechanisms that may alter the DM cornea. One such example would be targeting *LOX* in the KC cornea and increasing its expression such that LOX-mediated collagen-crosslinking is increased, thus resulting in corneal thickening. Additionally, by targeting inflammatory cascades such as the calpain/calpastatin system and the IL-1α/IL-1β to IL-1Ra ratio, we can then potentially halt the inflammatorymediated corneal thinning seen in KC. To conclude, by analyzing potential genetic associations in KC and DM, this review illustrates the areas where the current literature is lacking, with the hope of providing direction for future studies in elucidating the pathophysiology of KC and DM in the cornea. Future research would have tremendous benefit in identifying potential therapeutic targets in clinical management of KC.

## 9. Literature search

A comprehensive literature search completed by the end of September 2020 was performed on Pubmed. All selected articles were reviewed thoroughly by the authors to consolidate candidate genes that have been identified in genetic analyses and genome wide studies of keratoconus and central corneal thickness variations. We then explored how those respective genes may be similarly or differentially regulated under hyperglycemic conditions and the role they play in the systemic complications associated with diabetes.

## Figures and Tables

**Table 1:** List of human studies evaluating the relationship between keratoconus and diabetes (adapted and modified from ^26^).

Study	Study size	Design	Population Characteristics	Findings	Association

13	KC patients (n=571) Non-KC controls (n=571)	Retrospective case- control study	- German population - Hospital/clinic-based - Ethnicity unspecified - Age range: 20–40 yo; Mean age KC: 28.86 ± 5.79 years. Mean age control: 29.45 ± 5.75	T2DM showed a protective effect against KC development (odds ratio = 0.2195)	**Inverse association** of KC development with DM
14	KC patients without DM (n=269) KC patients with DM (n=26)	Retrospective cross-sectional study	- United States population - Wilmer Eye Institute; Hospital/ clinic-based - Ethnicity: White, Black, Other - Age range: 14–80 yo; Mean age 42.7 ± 13.4	T2DM showed a protective effect against more severe KC (odds ratio = 0.2); No difference in DM prevalence in KC population	**Inverse association** of DM with KC severity
17	KC patients (n=1383) non-KC controls (n=1383)	Retrospective case-control study	- Iranian population - Farabi Eye Hospital; Hospital/ clinic-based - Ethnicity unspecified - Age range: 18–49 yo. Mean age KC: 28.8 ± 5.3 years. Mean age control: 29.1 ± 5.8 years.	T2DM showed a protective effect against KC development (odds ratio = 0.350)	**Inverse association** of KC development with DM
18	KC patients (n=16,053) non-KC controls (n=16,053)	Retrospective longitudinal cohort study	- United States population - Population-based - Multiple ethnicity (White, Black, Latino, Asian, Other) - All ages included; Mean age 40.4 ± 13.0 years (KC and matched controls)	20% lower odds of KC development with uncomplicated DM; 52% lower odds of KC development with DM-associated organ failure	**Inverse association** of KC development with DM
19	KC patients (n=2679) non-KC controls (n=26,7900	Retrospective longitudinal cohort study	- Danish population - Population-based - Ethnicity: European vs. Non- European - All ages included. Mean age: 38.2 ± 15.9 (KC and matched controls)	No significant difference in DM prevalence in KC patients. Total DM odds ratio=1.03, T1DM odds ratio=0.87, T2DM odds ratio=1.07	**No significant association** between KC development and DM
298	KC patients (n=575) non-KC controls (n=2875)	Retrospective longitudinal cohort study	- Korean population - Population-based - Ethnicity unspecified - All ages included. Mean age: 31.1 ± 16.0 (KC and matched controls)	No significant difference in DM prevalence in KC patients. Multivariate odds ratio=1.02	**No significant association** between KC development and DM
299	29 studies incorporating 50,358,341 subjects	Systematic review and meta-analysis	- Global population; 15 countries - Hospital/clinic/population based	Odds of developing KC were 23% lower in T2DM, but relationship was not significant	**No significant association** between KC development and DM
22	KC patients (n=2051) non-KC controls (n=12,306	Retrospective case-control study	- Netherlands population - Population-based, comparable socioeconomic distribution - Relative age group 10–40 years. Mean age KC and control: 30 ± 6.5.	No significant association in KC and DM, with odds ratio 1.60 (0.89–2.89) and p-value 0.149.	**No significant association** between KC development and DM
20	KC patients (n=1377) non-KC controls (n=4131) AND T2DM KC patients (n=75) non-DM KC controls (n=225)	Retrospective case-control and Cross-sectional study	- United States population - Wills Eye Hospital Cornea Service; Hospital/clinic-based - Ethnicity unspecified - All ages included; Mean age KC: 44.64 ± 15.76 years. Mean age control: 45.06 ± 16.00	Higher prevalence of T2DM in KC population compared to controls (6.75% and 4.84%, respectively); Higher severity of KC in DM patients (odds ratio = 2.691)	**Positive association** of KC development with T2DM
21	KC patients (n=1552) non-KC controls (n=7.760)	Retrospective cohort study	- South Korean population - Population-based - Ethnicity unspecified - All ages included.	Higher prevalence of T2DM in KC population compared to controls (19.2% and 14.5%, respectively); Positive association of KC with DM (odds ratio = 1.35)	**Positive association** of KC development with DM

**Table 2:** List of genes that could mediate the potential correlation between keratoconus and diabetes.

Gene	Functions	CHR	KC/CCT effects	DM effects	References

**Cornea biomechanics and collagen crosslinking**					

*LOX*	Lysyl oxidase, participates in collagen crosslinking	5q23.2	Reduced LOX expression in corneal stroma and reduced activity in KC-derived corneal fibroblasts	Increased LOX expression and activity in retinal endothelial cells; unclear effect in DM cornea	33,45,47,58
*COL5A1*	Collagen type V, alpha-1 chain	9q34.2q34.3	COL5A1 haploinsufficiency results in corneal stroma thinning, reduced collagen fibers	Possible interaction between COL5A1 and HbA1c in DR study; no known direct effect on COL5A1 in cornea	65,66,72,81

**ECM remodeling**					

*FOXO1*	Transcription factor	13q14.1	SNP in FOXO1 linked to CCT, FOXO1 expression/activity unknown in cornea	FOXO1 linked to AGE-mediated disruption of autophagic flux and vascular endothelial cell autophagic apoptosis, role in cornea unknown	66,85,90
*SMAD3*	Transcription factor	15q22.23	SNP in SMAD3 linked to CCT, Increased pSMAD3 and increased TGFβ signaling in KC cells	SMAD3 linked to ECM remodeling in DN; role in cornea unclear	49,98,100,106
*TGFBI*	Transforming growth factor beta induced	5q31.1	SNP in TGFBI linked to KC with decreased levels of TGFBIp in KC cornea	Unknown effect in DM cornea, shown to be upregulated in response to high glucose and TGFβ in DM proximal tubules	124^–^126,132
*ZEB1*	Zinc finger transcription factor	10p11.22	Mutations in ZEB1 associated with KC and PPCD; possible genotype/phenotype correlation	Unknown effect in DM cornea, implicated in epithelial-to-mesenchymal transition under hyperglycemic conditions	152,154,155,158
*MMP-9*	Matrix metalloproteinase- 9	20q11.2q13.1	Increased MMP-9 activity noted in tear sample with corresponding upregulation in MMP-9 mRNA; SNP identified in MMP-9	Increased MMP-9 activity in tears from DM patients; SNP identified in MMP-9 associated with T2DM susceptibility	170,173,176,181,185
*TIMP-1*	Tissue inhibitor of metalloproteinases-1	Xp11.23	Decreased TIMP-1 levels detected in KC patients, SNP in TIMP-1 associated with increased KC risk	Increased TIMP-1 levels in tears of pediatric T1DM patients, but overall role of TIMP-1 in DM remains inconclusive	83,178,182,185
*MIR184*	microRNA	15q22q25	Mutations in miR-184 implicated in KC pathogenesis, but extent of association with KC alone remains unclear	miR-184 expression decreased in pancreatic β^–^cells in response to extracellular glucose; decreased in islet cells of T2DM patients	187,196,200,201,310,311

**Inflammation and ROS production**					

*HGF*	Hepatocyte growth factor	7q21.1	Increased HGF protein in KC corneal epithelium; increased HGF and c-Met mRNA in corneal wound healing	Increased HGF with decreased HGF receptor c-Met expression in DM cornea	3,214,217,220
*CAST*	Calpain/calpastatin, proteolytic degradation	5q15	SNP in CAST strongly linked to KC; CAST expression/activity unclear	High glucose induces calpain activity, increasing ROS production and vascular endothelial dysfunction; unknown effect in cornea	226,229,230
*SOD1*	Superoxide dismutase 1 cytoplasmic antioxidant enzyme	21q22.11	Deletion mutation in SOD1 in several cohorts; decreased SOD1 expression in KC corneal fibroblast cultures	Associated polymorphisms in SOD1 identified; decreased SOD1 expression in DM cornea with associated increase in RAGE	238^–^241,254,256
*IL1A/IL1B*	Interleukin 1alpha/beta, inflammatory cytokine	2q13	Increased IL-1α expression in KC corneas, SNPs identified in IL1A and IL1B	Imbalance in IL-1β to IL-1Ra in DM cornea, SNPs identified in IL1A, IL1B, and IL1RN in DM patients	119,162,203,262,268

**Additional genes of interest**					

*SPRY2*	Sprouty 2	13q31.1	SNP in SPRY2 linked to CCT and corneal epithelium proliferation; SPRY2 expression activity in KC cornea unknown	SNP near SPRY2 linked to increase DM susceptibility; unclear effect in SPRY2 cornea	49,278,280^–^282
*COL4A3, COL4A4*	Collagen type IV, alpha-3/4 chain, structural portion of corneal membranes	2q36.3	Alterations in collagen type IV reported in KC, but unclear if genetic polymorphisms play a role	Alterations in collagen type IV reported in DN and in the cornea under hypoxic conditions, unknown genetic association with DM cornea	48,283,290,291

## Abbreviations

ALS: amyotrophic lateral sclerosis
AGE: advanced glycation end product
CAST: calpastatin
CCT: central corneal thickness
CH: corneal hysteresis
CRF: corneal resistance factor
DM: diabetes mellitus
DN: diabetic nephropathy
DR: diabetic retinopathy
ECM: extracellular matrix
EDS: Ehler-Danlos syndrome
EMT: epithelial to mesenchymal transition
FECD: Fuch’s endothelial corneal dystrophy
FOXO1: forkhead box protein O1
GBM: glomerular basement membrane
GWAS: genome-wide association studies
HGF: hepatocyte growth factor
IHC: immunohistochemistry
KC: keratoconus
lncRNA: long noncoding RNA
LOX: lysyl oxidase
miRNA: microRNA
MMP: matrix metalloproteinases
PPCD: posterior polymorphous corneal dystrophy
RAGE: receptor for advanced glycation end products
SOD1: superoxide dismutase 1
TIMP: tissue inhibitors of metalloproteinases
TGFBI: transforming growth factor beta-induced
TGFBIp: transforming growth factor beta-induced protein
VSX1: visual system homeobox 1
ZEB1: zinc finger E box-binding homeobox 1
